# An Introduction to Stochastic Deep Learning

**DOI:** 10.1002/wics.70076

**Published:** 2026-09-19

**Authors:** Faming Liang

**Affiliations:** Department of Statistics, Purdue University, West Lafayette, Indiana, USA

## Abstract

Deep neural networks (DNNs) have achieved remarkable success in prediction, but their deterministic formulation makes many statistical inference tasks difficult. StoNet, short for stochastic neural network, addresses this limitation by reformulating a DNN as a probabilistic latent-variable model, in which the outputs of selected hidden layers or units are treated as latent variables. The article describes the formulation of StoNet and its asymptotic equivalence to a conventional DNN, showing how StoNet can serve both as a deep learning model and as an analytical device for studying DNNs. It reviews training algorithms for StoNet and discusses its applications to statistical inference problems, including nonlinear sufficient dimension reduction, causal inference with observed and unobserved confounders, Granger-causality learning for nonlinear time series, nonlinear variable selection, and prediction uncertainty quantification for large-scale DNNs. The article also discusses two related architectures: kernel-expanded StoNet and sublinearly structured DNNs, which can be viewed as structural or theoretical extensions of the StoNet framework. Under suitable architectural conditions, the former is immune to local traps in training and tends to outperform conventional DNNs in prediction; whereas the latter achieves feature-learning consistency in over-parameterized regimes when the training sample size is sufficiently large. Together, these developments show that StoNet broadens deep learning from prediction-oriented function approximation to a richer framework for statistical inference.

## 1 | Introduction

Deep learning has revolutionized modern data science, driving major breakthroughs over the past decade in areas such as image generation, protein folding, and natural language processing. From a statistical perspective, however, deep learning generally lacks the inferential capabilities traditionally expected of statistical models. In particular, it is often unclear how to perform uncertainty quantification, sufficient dimension reduction, model selection, and causal effect estimation using DNNs. These difficulties are largely due to the complex architectures of DNNs and their predominantly deterministic nature as function approximators.

Motivated by the fundamental importance of statistical inference in modern data science, Liang and collaborators introduced the stochastic neural network framework known as StoNet by randomizing selected hidden units or hidden layers of traditional DNNs (Liang et al. 2022; Sun and Liang 2022, 2027; Fang and Liang 2024, 2026). In this article, we use the term StoNet to refer specifically to this latent-variable formulation of DNNs. The added stochasticity changes the role of neural networks from purely deterministic function approximators into probabilistic models for complex systems. Specifically, traditional neural networks can be viewed as compositions of affine transformations and nonlinear activations, whereas StoNets can be viewed as compositions of linear, nonlinear, or logistic regression modules. This probabilistic formulation makes it possible to develop statistical inference tools for complex systems.

In addition to StoNet, several important classes of stochastic neural networks have appeared in the literature, including energy-based models, Bayesian neural networks, dropout networks, variational autoencoders, and related architectures. Energy-based models include Boltzmann machines (BMs) (Ackley et al. 1985), restricted Boltzmann machines (RBMs) (Smolensky 1986; Hinton 2002), and deep belief networks (Hinton et al. 2006). These models generally define joint distributions over visible and hidden units and have served as useful tools for extracting features from visible data. Historically, such feature-extraction methods provided an effective way to initialize deep architectures (Hinton and Salakhutdinov 2006; Salakhutdinov and Hinton 2009) before purely supervised training became reliably effective, thereby contributing significantly to the development of modern deep learning techniques. Bayesian neural networks (MacKay 1996; Neal 1996; Liang 2005) have long served as flexible tools for Bayesian modeling of complex systems, while Bayesian sparse neural networks (Liang, Li, and Zhou 2018; Sun et al. 2022a) provide a fundamental framework for nonlinear variable selection. Dropout networks (Hinton et al. 2012; Gal and Ghahramani 2016) randomly mask hidden units or connections during training and can be interpreted as approximate Bayesian models with Bernoulli-type stochasticity imposed on hidden units or connections. Variational autoencoders (Kingma and Welling 2014) incorporate stochastic latent layers for generative modeling. In addition, some studies have improved robustness and generalization of DNNs through stochastic activations, see for example, Gülçehre et al. (2016), Noh et al. (2017), You et al. (2018), Yu et al. (2021).

This article provides a brief review of stochastic deep learning, with a particular focus on the theory, computation, and applications of StoNet developed by Liang and collaborators. We begin with the mathematical formulation of StoNet, describing its asymptotic equivalence to a conventional DNN and its bridging role in extending statistical learning theory from linear models to DNNs. Next, we review training algorithms for StoNet, including those based on the imputation-regularized optimization (IRO) algorithm (Liang, Jia, et al. 2018) and adaptive stochastic-gradient MCMC algorithms (Deng et al. 2019; Liang et al. 2022). We then discuss important applications of StoNet in statistical inference, including sufficient dimension reduction (Liang et al. 2022), causal inference (Fang and Liang 2024, 2026), Granger causality (Ahn et al. 2026), and uncertainty quantification for large-scale DNNs (Sun and Liang 2027). Finally, we discuss two related architectures: kernel-expanded StoNet (Sun and Liang 2022) and sublinearly structured DNNs (Kim et al. 2026), which can be viewed as structural or theoretical extensions of the StoNet framework. The former illustrates how kernel methods and DNNs can be integrated to accelerate training and improve generalization, while the latter shows how StoNet can serve as a bridge for understanding the success of DNNs and for guiding the design of neural network architectures. In particular, sublinear DNNs achieve feature-learning consistency in over-parameterized regimes when the training sample size is sufficiently large.

## 2 | Stochastic Neural Networks

### 2.1 | Mathematical Formulation

The StoNet is a probabilistic analogue of a DNN in which selected hidden-layer outputs are modeled as latent random variables. A basic Gaussian StoNet, see Figure 1 for an illustration, can be written as follows:

(1) $$Y_{1}=g_{1}\left(X,b_{1},W_{1}\right)+e_{1},\;Y_{i}=g_{i}\left(\Psi \left(Y_{i-1}\right),b_{i},W_{i}\right)+e_{i},\;i=2,\ldots ,h,\;Y=g_{h+1}\left(\Psi \left(Y_{h}\right),b_{h+1},W_{h+1}\right)+e_{h+1},$$

where $Y_{1},\ldots ,Y_{h}$ are latent representations, $e_{i}\sim N\left(0,\sigma_{i}^{2}I_{d_{i}}\right)$, and $g_{i}(\cdot )$ denotes the neural network function for module i. For theoretical simplicity, one may take all module functions $g_{i}$’s to be linear, corresponding to modules with a single hidden layer; see Liang et al. (2022) and Sun and Liang (2027) for details. For classification, the output layer can be replaced by a binary or multiclass logistic regression, with $\sigma_{h+1}^{2}$ interpreted as an output-layer temperature.^1^ The model may be viewed as a composition of linear, nonlinear, or logistic regressions, making the architecture more interpretable and modular. Throughout the paper, we let $\theta_{i}=\left(b_{i},W_{i}\right),i=1,\ldots ,h+1$, denote the parameters of module i, and let $\theta =\left(\theta_{1},\ldots ,\theta_{h+1}\right)$ denote the full collection of model parameters. Also, we write $Y_{\text{mis}}=\left(Y_{1},\ldots ,Y_{h}\right)$ for the collection of latent hidden-layer variables. Since these variables are unobserved, likelihoods involving $\left(Y,Y_{\text{mis}}\right)$ should be understood as complete-data objectives induced by the StoNet latent-variable formulation. In practice, such objectives are approximated through sampling or imputation, as discussed in Section 3.

The StoNet can be viewed as a statistical latent-variable model. By introducing appropriate stochastic layers, it enables uncertainty propagation, decomposes large networks into smaller modules, and supports local computation as well as joint modeling of multiple complex systems. When the latent variable noise is appropriately controlled, the induced learning objective is asymptotically equivalent to that of the corresponding conventional DNN; see Section 2.2. These and related properties will be discussed in detail in the remainder of this article.

### 2.2 | Asymptotic Equivalence Between StoNet and DNN

For convenience, we write the conventional DNN corresponding to the StoNet(1), with deterministic hidden-layer propagation and observational error only at the output layer, as follows:

(2) $$Y_{1}=g_{1}\left(X,b_{1},W_{1}\right),\;Y_{i}=g_{i}\left(\Psi \left(Y_{i-1}\right),b_{i},W_{i}\right),\;i=2,\ldots ,h,\;Y=g_{h+1}\left(\Psi \left(Y_{h}\right),b_{h+1},W_{h+1}\right)+e_{h+1},$$

where $e_{h+1}\sim N\left(0,\sigma_{h+1}^{2}I_{d_{h+1}}\right)$ are random errors contained in observations. To formalize StoNet as a valid surrogate for the DNN in (2)—in the sense that their induced loss functions coincide asymptotically—we first consider the case in which all $g_{i}$’s are linear, and then extend the results to the modular setting. To this end, we impose the following regularity conditions. Let Θ denote the parameter space of StoNet, and let $d_{\theta }$ denote the dimension of θ. Let $\tilde{h}$ denote the total number of hidden layers, including both stochastic and deterministic layers, of the StoNet.

Assumption 1.

- (Compactness of parameter space) The parameter space Θ is compact; specifically, Θ is contained in a $d_{\theta }$-dimensional Euclidean ball centered at 0 with radius r.
- (Log-likelihood square-integrability) $E\{\text{log}\pi (Y|X,\theta ){\}}^{2}<\infty$ for every θ∈Θ.
- (Lipschitz activation functions) The activation Ψ(·) is $c^{\prime }$-Lipschitz for some constant $c^{\prime }>0$.
- (Growing architecture) Both the depth $\tilde{h}$ and the layer widths $\left\{d_{k}\right\}$ are allowed to increase with n.
- (Layerwise noise scaling) The noise added to the hidden layers satisfies the conditions: the output layer noise scale $\sigma_{h+1}=O(1)$; and for each random hidden layer $k\in \{1,2,\ldots ,\tilde{h}\}$, the nose scale $\sigma_{k}$ is chosen such that $\sigma_{k}≺1/d_{k}$ and
(3) $$d_{\tilde{h}+1}\left(\prod_{i=k+1}^{\tilde{h}}d_{i}^{2}\right)d_{k}\sigma_{k}^{2}≺\frac{1}{\tilde{h}}.$$

Conditions (i)–(iv) are mild regularity assumptions commonly used in theoretical analyses of DNNs and are typically met in practical applications. Condition(v) regulates the scale of the noise introduced at the hidden layers, whose dependence on the training sample size n is implicit. The factor $d_{\tilde{h}+1}\left(\prod_{i=k+1}^{\tilde{h}}d_{i}^{2}\right)d_{k}$ reflects the extent to which the perturbation $e_{k}$ may be magnified as it passes through the remaining layers and reaches the output. Condition (3) requires this propagated noise contribution to be asymptotically negligible. Together with the assumption $\sigma_{k}≺1/d_{k}$, this condition provides control over the eigenvalues of each hidden layer (Kim et al. 2026), which in turn enables a rigorous structural analysis of DNNs. Notably, these conditions should be regarded as convenient sufficient conditions for Theorem 1, rather than as minimal assumptions. A sharper characterization of the necessary and sufficient conditions remains an interesting direction for future research.

Theorem 1 shows that the StoNet and the corresponding DNN are asymptotically equivalent in their training losses: As the training sample size becomes large, the training objectives induced by treating the hidden-layer outputs as latent variables and as a deterministic forward map become indistinguishable.

**Theorem 1. (Theorem 2.1 of** Liang et al. 2022**).** *Suppose that all module functions* $g_{i}$’*s are linear and Assumption 1 holds. Then the StoNet* (1) *and the DNN* (2) *share the same loss function asymptotically, in the sense that*

(4) $$\underset{\theta \in \Theta }{\text{sup}}\left|\frac{1}{n}\sum_{i=1}^{n}\text{log}\;\pi \left(Y^{\left(i\right)},Y_{\text{mis}}^{\left(i\right)}|X^{\left(i\right)},\theta \right)-\frac{1}{n}\sum_{i=1}^{n}\text{log}\;\pi \left(Y^{\left(i\right)}|X^{\left(i\right)},\theta \right)\right|\overset{p}{\to }0,$$

*as* n→∞.

*Remark* 1. (*Extension to modular StoNets*). Theorem 1 is stated for the case in which the module functions $g_{i}$ are linear. This restriction is mainly for notational simplicity. The same argument extends naturally to the modular StoNet in (1), where each ${\text{g}}_{\text{i}}\left(\cdot ,b_{\text{i}},W_{\text{i}}\right)$ is itself a small neural-network module. Indeed, each such module can be expanded into a finite composition of affine transformations and nonlinear activations. After this expansion, the modular StoNet can be viewed as an ordinary StoNet with additional layers. The layers introduced by the expansion are deterministic, so their noise variances can be set to zero, or equivalently treated as asymptotically negligible, without violating the noise-scaling requirements in Assumption 1. Consequently, the complete-data StoNet objective and the corresponding conventional DNN objective remain asymptotically equivalent for modular StoNets.

Define the population objective for the conventional DNN:

(5) $$Q^{*}(\theta )=E_{\pi (X,Y)}[\text{log}\;\pi (Y|X,\theta )]$$

where the expectation is with respect to the joint distribution of (X,Y). Under Assumption 1(i)–(ii), a uniform law of large numbers gives,

(6) $$\underset{\theta \in \Theta }{\text{sup}}\left|\frac{1}{n}\sum_{i=1}^{n}\text{log}\;\pi \left(Y^{\left(i\right)}|X^{\left(i\right)},\theta \right)-Q^{*}(\theta )\right|\overset{p}{\to }0.$$

To guarantee a well-defined population objective, we impose the following additional regularity assumption on $Q^{*}(\theta )$.

Assumption 2.

- The function $Q^{*}(\theta )$ is continuous on Θ and admits a single maximizer, denoted by $\theta^{*}$.
- For every ϵ>0, let $B(\epsilon )=\left\{\theta :\left\|\theta -\theta^{*}\right\|<\epsilon \right\}$ denote a small neighborhood around $\theta^{*}$. Then ${\text{sup}}_{\theta \in \Theta \setminus B(\epsilon )}Q^{*}(\theta )$ exists and satisfies $Q^{*}\left(\theta^{*}\right)-{\text{sup}}_{\theta \in \Theta \setminus B(\epsilon )}Q^{*}(\theta )>0$.

**ALGORITHM 1 |:** Adaptive SGHMC for StoNet Training (Liang et al. 2022).

1: **Input:** total iterations T; inner SGHMC steps $k_{\text{HMC}}$; learning rates ${\left\{\epsilon_{t}\right\}}_{t=1}^{T}$; step sizes ${\left\{\gamma_{t}\right\}}_{t=1}^{T}$; dataset D with sample size n.
2: **Initialize:** $\theta_{0},Y_{\text{mis}}^{\left(0\right)}$ and $V^{\left(0\right)}$.
3: **for** t=0,1,…,T-1 **do**
4: **Step 0 (Subsample).** Draw a mini-batch of size $n_{b}$ from 𝒟.
5: **Step 1 (Sampling).** Run $k_{\text{HMC}}$ SGHMC steps starting from $Y_{\text{mis}}^{\left(t+1,0\right)}=Y_{\text{mis}}^{\left(t\right)}$ and $V^{\left(t+1,0\right)}=V^{\left(t\right)}$:
6: **for** $\ell =0,1,\ldots ,k_{\text{HMC}}-1$ **do**
(8) $$V^{\left(t+1,\ell +1\right)}=\left(1-\epsilon_{t+1}\eta \right)V^{\left(t+1,\ell \right)}+\epsilon_{t+1}\nabla \tilde{L}\left(\theta_{t},Y_{\text{mis}}^{\left(t+1,\ell \right)}\right)+\sqrt{2\tau \eta \epsilon_{t+1}}e_{t+1}^{\left(\ell +1\right)},$$
$$Y_{\text{mis}}^{\left(t+1,\ell +1\right)}=Y_{\text{mis}}^{\left(t+1,\ell \right)}+\epsilon_{t+1}V^{\left(t+1,\ell \right)},$$
where $e_{t+1}^{\left(\ell +1\right)}\sim N\left(0,I_{d_{\text{mis}}}\right),d_{\text{mis}}$ denotes the dimension of $Y_{\text{mis}}$, and $\nabla \tilde{L}\left(\theta_{t},Y_{\text{mis}}^{\left(t+1,\ell \right)}\right)$ denotes an unbiased estimator of the gradient of $\text{log}\;\pi \left(Y_{\text{mis}}|\theta ,X,Y\right)$ with respect to $Y_{\text{mis}}$.
7: **end for**
8: Set $Y_{\text{mis}}^{\left(t+1\right)}=Y_{\text{mis}}^{\left(t+1,k_{\text{HMC}}\right)}$ and $V^{\left(t+1\right)}=V^{\left(t+1,k_{\text{HMC}}\right)}$.
9: **Step 2 (Parameter update)**.
$$\theta_{t+1}=\theta_{t}+\gamma_{t+1}H\left(Y_{\text{mis}}^{\left(t+1\right)},\theta_{t}\right).$$
10: **end for**
11: **Output:** ${\left\{\left(Y_{\text{mis}}^{\left(t\right)},\theta_{t}\right)\right\}}_{t=1}^{T}$.

Assumption 2 is a technical identifiability condition: it ensures that the population objective is well behaved and that $\theta^{*}$ is identifiable as its unique maximizer. Strictly speaking, $\theta^{*}$ is identifiable only up to loss-invariant transformations, such as reordering hidden neurons within the same hidden layer or simultaneously altering the sign or scale of certain connection weights. Accordingly, $\theta^{*}$ can be treated as unique in theoretical studies, with the understanding that it represents a canonical member of the equivalence class of parameterizations that induce the same network function, and hence the same objective. The following consistency result concerns the complete-data StoNet objective. Since $Y_{\text{mis}}$ is unobserved, this objective is implemented in practice through imputation or sampling from the conditional distribution of the latent variables, as described in Section 3.

**Theorem 2. (Theorem 2.2 of** Liang et al. 2022**).** *Assume that Assumptions 1 and 2 are satisfied, and that* $\pi \left(Y,Y_{\text{mis}}|X,\theta \right)$ *is continuous in θ*. *Let* ${\hat{\theta }}_{n}$ *denote a maximizer of the complete-data StoNet objective, that is*

$${\hat{\theta }}_{n}\in arg\underset{\theta \in \Theta }{max}\left\{\frac{1}{n}\sum_{i=1}^{n}log\pi \left(Y^{\left(i\right)},Y_{mis}^{\left(i\right)}|X^{\left(i\right)},\theta \right)\right\}.$$

*Then*

$$\left\|{\hat{\theta }}_{n}-\theta^{*}\right\|\overset{p}{\to }0,\;n\to \infty .$$

These conclusions justify using the StoNet formulation (1) as a training surrogate for the conventional DNN (2). As the training sample size increases, the StoNet and its corresponding DNN induce asymptotically equivalent learning objectives. Moreover, the maximizer of the StoNet objective converges to the same population target in the large-sample limit.

## 3 | Training Algorithms

Under the missing-data framework, Theorems 1 and 2 imply that training a StoNet can be formulated as solving the meanfield equation

(7) $$E\left[H\left(Y_{\text{mis}},\theta \right)\right]=\int H\left(Y_{\text{mis}},\theta \right)\pi \left(Y_{\text{mis}}|\theta ,X,Y\right)dY_{\text{mis}}=0,$$

where $H\left(Y_{\text{mis}},\theta \right)=\nabla_{\theta }\text{log}\;\pi \left(Y,Y_{\text{mis}}|X,\theta \right)$. This equation can be solved using an adaptive stochastic gradient Markov chain Monte Carlo (SG-MCMC) algorithm that alternates between the following two steps at iteration t:

- *Latent-variable sampling*: sample $Y_{\text{mis}}^{\left(t+1\right)}$ from a transition kernel whose invariant distribution is $\pi \left(Y_{\text{mis}}|\theta^{\left(t\right)},X,Y\right)$.
- *Parameter updating*: update $\theta^{\left(t\right)}$ to $\theta^{\left(t+1\right)}$ using a stochastic-gradient step based on the current latent draw $Y_{\text{mis}}^{\left(t+1\right)}$.

Adaptive SG-MCMC algorithms, such as adaptive SGLD (Deng et al. 2019) and adaptive SGHMC (Liang et al. 2022), can be applied directly to solving (7). In particular, Liang et al. (2022) employed adaptive SGHMC; see Algorithm 1. Under suitable regularity conditions, they established the consistency of $\theta^{\left(t\right)}$ for $\theta^{*}$, as well as weak convergence of $Y_{\text{mis}}^{\left(t\right)}$ to $\pi \left(y_{\text{mis}}|\theta^{*},X,Y\right)$. These results provide a theoretical foundation for statistical inference with StoNet.

More precisely, Liang et al. (2022) recommended choosing the learning rate and step size as

(9) $$\epsilon_{k}=\frac{C_{\epsilon }}{c_{e}+k^{\alpha }},\;\gamma_{k}=\frac{C_{\gamma }}{c_{g}+k^{\alpha }},$$

where $\alpha \in (0,1),C_{e},C_{\gamma }>0$, and $c_{e},c_{g}\geq 0$. They first proved the parameter convergence rate

(10) $$E\left[{\left\|\theta_{t}-\theta^{*}\right\|}^{2}\right]\leq \zeta \gamma_{t},$$

for some constant ζ>0, and then controlled the Wasserstein distance between the time-t law of $Y_{\text{mis}}^{\left(t\right)}$, denoted by $\mu_{t}=ℒ\left(y_{\text{mis}}^{\left(t\right)}|\theta_{t}\right)$, and the corresponding continuous-time target $\pi^{*}=\pi_{\theta^{*}}\left(y_{\text{mis}}\right)$. Specifically, they established the following 2-Wasserstein bound:

(11) $${𝒲}_{2}\left(\mu_{t},\pi^{*}\right)\leq C_{0}\sqrt{{ℋ}_{\rho }\left(\mu_{0},\pi^{*}\right)}e^{-\lambda_{*}T_{t}}+\sqrt{C_{5}\text{log}\;T_{t}}\left\{\sqrt{\tilde{C}(t)}+{\left(\frac{\tilde{C}(t)}{2}\right)}^{1/4}\right\}+\sqrt{C_{6}T_{t}\sum_{j=1}^{t-1}\epsilon_{j+1}^{2}},$$

where $T_{t}=\sum_{j=0}^{t-1}\epsilon_{j+1}$, and $\tilde{C}(t)=C_{1}T_{t}^{2}\sum_{j=1}^{t-1}\epsilon_{j+1}^{2}+C_{2}\sum_{j=1}^{t-1}\epsilon_{j+1}\gamma_{j}+C_{3}\delta_{g}T_{t}+C_{4}\sum_{j=1}^{t-1}\epsilon_{j+1}^{2}$. Here $C_{0},\ldots ,C_{6}>0$ and $\lambda_{*}>0$ are constants, and ${ℋ}_{\rho }\left(\mu_{0},\pi^{*}\right)$ is an ρ-semimetric on probability measures; see, for example, Gao et al. (2021).

For a fixed time horizon $T_{t}$, the second and third terms in (11) can be made arbitrarily small by choosing sufficiently small initial learning rate $\epsilon_{1}$ and step size $\gamma_{1}$, and by taking the mini-batch size $n_{b}$ sufficiently large. In practice, however, these choices must be balanced against computational cost and the need to let $T_{t}\to \infty$ over time, so that the exponential term $e^{-\lambda_{*}T_{t}}$ also vanishes.

When all module function are taken to be linear, StoNet can also be trained using the stochastic EM algorithm (Celeux and Diebolt 1985; Nielsen 2000) or its high-dimensional extension, the imputation-regularized optimization algorithm (Liang, Jia, et al. 2018), see Algorithm 2 for the case with a sparse Lasso penalty. Although these algorithms may be inefficient for large networks, their mathematical tractability makes them useful for establishing key properties of StoNet in specific applications, as discussed later. In addition, these algorithms allow *local computation*: conditional on the imputed latent variables, one may update only a subset of θ. This locality makes StoNet learning conceptually related to biologically plausible computation.

## 4 | Applications of StoNets in Modern Data Science

This section presents several classes of applications of StoNets in modern data science, including sufficient dimension reduction, causal inference, nonlinear variable selection, and prediction uncertainty quantification for large-scale DNNs.

### 4.1 | Sufficient Dimension Reduction

Sufficient dimension reduction (SDR) is a supervised representation-learning framework that seeks to replace high-dimensional predictors with a lower-dimensional representation while preserving all predictor information relevant to the response. Let $Y\in R^{d}$ denote the response and let $X={\left(X_{1},\ldots ,X_{p}\right)}^{⊤}\in R^{p}$ denote the covariates. Mathematically, SDR aims to construct a reduced representation $Z\in R^{q}$, with q<p, as a function of X, such that the conditional distribution of Y given X depends on X only through Z; that is,

(12) P(Y∣X)=P(Y∣Z),equivalentlyY⫫X∣Z,

where ⫫ denotes conditional independence. Thus, Z serves as a sufficient summary of X for modeling or predicting Y.

The SDR literature includes both linear and nonlinear methods for addressing this conditional-independence objective. In the linear setting, it seeks a matrix $B\in R^{p\times q}$ such that

(13) $$Y⫫X|B^{⊤}X.$$

Many methods have been developed to estimate such a subspace, including inverse regression methods (Li 1991; Cook 2000; Cook and Weisberg 1991; Bura and Cook 2001; Li et al. 2005; Li and Wang 2007) and forward regression methods (Li 1992; Xia et al. 2002; Fertl and Bura 2022). In the nonlinear setting, SDR instead seeks a nonlinear representation f(X) such that

(14) Y⫫X∣f(X).

One common strategy is to kernelize linear SDR methods by mapping X into a feature space through a kernel and then applying inverse or forward SDR techniques in that space (Wu 2008; Fukumizu et al. 2009; Fukumizu and Leng 2014; Nilsson et al. 2007; Lee et al. 2013; Suzuki and Sugiyama 2010). Another line of work studies nonlinear SDR through multi-index models and related constructions (Xia 2007; Hristache et al. 2001). More recently, neural network-based SDR methods have also been developed; see, for example, Liang et al. (2021) and Tang and Li (2026).

StoNet provides a principled framework for nonlinear SDR. Owing to its layer-wise Markov structure, the joint conditional distribution of the response and the hidden-layer representations factorize as follows:

(15) $$\pi \left(Y,Y_{\text{mis}}|X,\theta \right)=\pi \left(Y_{1}|X,\theta_{1}\right)\left\{\prod_{i=2}^{h}\pi \left(Y_{i}|Y_{i-1},\theta_{i}\right)\right\}\pi \left(Y|Y_{h},\theta_{h+1}\right).$$

Consequently, $\pi \left(Y|Y_{\text{mis}},X,\theta \right)=\pi \left(Y|Y_{h},\theta_{h+1}\right)$ holds, which further implies

(16) $$Y⫫X|Y_{h},\theta .$$

This result can be summarized in the following lemma.

**Lemma 1. (Proposition 2.1 of** Liang et al. 2022). *For a well-trained StoNet model describing the conditional relationship between* X *and* Y, *the last hidden-layer representation* $Y_{h}$ *provides a nonlinear sufficient dimension reduction of* X *with respect to* Y; *that is*, (16) *holds*.

Lemma 1 identifies StoNet as a flexible and theoretically grounded tool for nonlinear SDR. Although an analogous conditional-independence relation may also be formulated for intermediate hidden layers, Liang et al. (2022) focus on the last hidden layer to avoid the identifiability issues that may arise from the universal approximation capability of DNNs. Moreover, by the asymptotic equivalence between the StoNet and the corresponding DNN, Lemma 1 further suggests that, as n→∞, hidden-layer representations of the corresponding conventional DNN can also inherit the SDR property. This observation is consistent with the belted and ensembled neural network framework of Tang and Li (2026), in which a narrow latent layer, called the belt, is deliberately inserted to extract a low-dimensional SDR representation.

In their numerical study, Liang et al. (2022) compared StoNet with several nonlinear SDR methods, including generalized sliced inverse regression (GSIR) (Lee et al. 2013), generalized sliced average variance estimator (GSAVE) (Lee et al. 2013), and an autoencoder baseline, on a sub-MNIST dataset. Their results show that StoNet produces more discriminative low-dimensional representations than the competing methods. Their study also highlights the computational scalability of StoNet relative to kernel-based nonlinear SDR methods such as GSIR and GSAVE.

### 4.2 | Causal Inference

Consider the problem of causal inference under the potential-outcomes framework (Rubin 1974). Let A∈{0,1} denote the treatment indicator, X the observed confounders, and Y(a) the potential outcome under treatment level a∈{0,1}. The average treatment effect (ATE) is defined as

τ=E{Y(1)-Y(0)}.

Identification of the ATE typically relies on the conditions: ignorability, overlap, and stable unit treatment value assumption (SUTVA), as stated in Assumption 3. Classical ATE estimation methods include outcome regression, inverse probability weighting, augmented inverse probability weighting (AIPW), and matching; see, for example, Imbens (2004), Rosenbaum (2002), and Robins et al. (1994).

Assumption 3.

- (*Ignorability*) {Y(0),Y(1)}⫫A∣X.
- (*Overlap*) $0<\zeta_{1}\leq P(A=1|X)\leq \zeta_{2}<1$ almost surely, for some constants $\zeta_{1},\zeta_{2}$.
- (*SUTVA*) The potential outcome of one unit is unaffected by the treatment assignment of any other unit.

In plain terms, ignorability requires that, after conditioning on the observed covariates X, treatment assignment is effectively independently of the potential outcomes; that is, there are no remaining unobserved confounders that jointly affect treatment assignment and the potential outcomes. The overlap condition requires that, for every covariate profile occurring in the population, each treatment level has a positive probability of being assigned. This condition ensures that the treatment effect can be learned by comparing treated and untreated units throughout the covariate space. Finally, SUTVA requires that the potential outcome of each unit depend only on that unit’s own treatment assignment, thereby ruling out interference or spillover effects between units. Together, these conditions enable identification of the ATE from the observed-data distribution.

For the class of complex causal problems considered in this article, the stochastic formulation of StoNet is more than a computational device: it provides the probabilistic structure needed to represent latent quantities that commonly arise in real data. Such quantities may include missing covariates, unobserved confounders, latent states, or intermediate biological and behavioral variables. A conventional DNN represents its hidden layers as deterministic transformations and therefore does not, by itself, preserve the randomness of these latent quantities. In contrast, the stochastic layers or units of StoNet treat hidden representations as random variables, allowing them to be imputed, integrated out, and propagated through the network. Moreover, the layerwise Markov structure of StoNet induces conditional-independence relationships that factorize a complex joint distribution into simpler modules. These conditional-independence structures are particularly important for causal inference, where identification and estimation rely on separating the treatment-assignment mechanism, the outcome mechanism, and, when needed, the distribution of latent confounders. StoNet therefore provides a natural framework for translating a causal graph into a trainable probabilistic neural network while retaining the latent-variable and conditional-independence structures required for statistical inference.

#### 4.2.1 | Causal StoNet With Observed Confounders

Causal-StoNet (Fang and Liang 2024), with its architecture depicted in Figure 2, embeds the treatment assignment into an intermediate layer of a StoNet architecture. This illustrates a broader modeling capability of StoNet: it can incorporate variables or observations that arise at intermediate stages of a complex system, such as treatment assignment in causal inference or gene expression in SNP–gene expression–disease modeling. The architecture can also naturally accommodate missing covariate values, as illustrated in Figure 2. In the observed-confounder setting considered here, however, all confounders are assumed to be observed.

**ALGORITHM 2 |:** IRO Algorithm for Training a StoNet With Linear Modules and Lasso Penalty (Sun and Liang 2027).

1: **Input:** Dataset D=(Y,X) of sample size n, total number of iterations T, and the number of Monte Carlo updates” $t_{MC}\text{.}$
2: **Initialization:** Generate an initial value for the network parameters ${\overset{ˆ}{\theta }}^{\left(0\right)}=\left({\overset{ˆ}{\theta }}_{1}^{\left(0\right)},\ldots ,{\overset{ˆ}{\theta }}_{h+1}^{\left(0\right)}\right)$.
3: **for** t=1,2,…,T **do**
4: **(i) Imputation:** For each observation $\left(X^{\left(i\right)},Y^{\left(i\right)}\right)$, sample $Y_{\text{mis}}^{\left(i,t\right)}$ from
$$\pi \left(Y_{\text{mis}}^{\left(i\right)}|Y^{\left(i\right)},X^{\left(i\right)},{\overset{ˆ}{\theta }}^{\left(t-1\right)},\sigma_{n}^{2}\right)$$
by applying a Langevin-dynamics transition kernel for $t_{\text{MC}}$ steps.
5: **(ii) Penalized parameter update:** Based on the pseudo-complete data $\left\{\left(Y^{\left(i\right)},Y_{\text{mis}}^{\left(i,t\right)},X^{\left(i\right)}\right):i=1,\ldots ,n\right\}$, update the parameters layer by layer:
6: **for** l=1,2,…,h+1 **do**
7: Compute
(24) $${\hat{\theta }}_{l}^{\left(t\right)}=\text{arg}\;\underset{\theta_{l}}{\text{min}}\left\{-\frac{1}{n}\overset{n}{\underset{i=1}{\sum }}\text{log}\;\pi \left(Y_{l}^{\left(i,t\right)}|Y_{l-1}^{\left(i,t\right)},\theta_{l},\sigma_{l}^{2}\right)+P_{\lambda_{n}}\left(\theta_{l}\right)\right\},$$
by solving a multivariate linear or logistic regression problem, where h denotes the number of hidden layers, $Y_{h+1}^{\left(i,t\right)}=Y^{\left(i\right)},Y_{0}^{\left(i,t\right)}=X^{\left(i\right)}$, and $\theta_{l}=\left\{W_{l},b_{l}\right\}$ represents the parameters of layer l.
8: **end for**
9: **end for**
10: **Output:** The final parameter estimate ${\overset{ˆ}{\theta }}^{\left(T\right)}=\left({\overset{ˆ}{\theta }}_{1}^{\left(T\right)},\ldots ,{\overset{ˆ}{\theta }}_{h+1}^{\left(T\right)}\right)$.

In Causal-StoNet, the joint distribution $\pi \left(Y,Y_{\text{mis}},A|X,\theta \right)$ can be factorized as follows:

(17) $$\pi \left(Y,Y_{\text{mis}},A|X,\theta \right)=\pi \left(Y_{1}|X,\theta_{1}\right)\pi \left(Y_{2}|Y_{1},\theta_{2}\right)\pi \left(A|Y_{1},\theta_{2}\right)\times \pi \left(Y_{3}|Y_{2},A,\theta_{3}\right)\pi \left(Y|Y_{3},\theta_{4}\right).$$

This factorization allows the propensity score and outcome regression function to be estimated jointly. Specifically, the treatment module $\pi \left(A|Y_{1},\theta_{2}\right)$ plays the role of the propensity score model, whereas the output module models the outcome response. To handle high-dimensional covariates, Fang and Liang (2024) imposed a sparsity-inducing mixture-Gaussian prior on the network weights and biases,

$$\theta \sim \lambda_{n}N\left(0,\sigma_{1,n}^{2}\right)+\left(1-\lambda_{n}\right)N\left(0,\sigma_{0,n}^{2}\right),$$

where θ represents a generic component of $\theta ,\sigma_{0,n}^{2}$ is small, and $\sigma_{1,n}^{2}$ is comparatively large.

Under suitable regularity and sparsity conditions, Causal-StoNet consistently estimates both the propensity score and the outcome regression function, and also consistently recovers the sparse network structure. Plugging the estimated propensity and outcome models into the augmented inverse probability weighted (AIPW) formula (Robins et al. 1994) yields an estimator of the ATE, which inherits the usual double-robust property when either the propensity score model or the outcome regression model is correctly specified. Moreover, because the propensity and outcome models are learned jointly, covariates selected for the propensity model are naturally embedded in the broader outcome-relevant architecture, which facilitates interpretation of selected covariates.

In applications with observed confounders, Fang and Liang (2024) compared Causal-StoNet with several existing ATE estimation methods. Their results suggest that the joint StoNet modeling of treatment assignment and outcome response produces stable ATE estimates and competitive uncertainty quantification, supporting the usefulness of the architecture for high-dimensional causal adjustment.

When missing values are present in some covariates, they can be imputed jointly with the latent variables $Y_{\text{mis}}$ at each iteration of the adaptive SG-MCMC algorithm. This extension broadens the applicability of Causal-StoNet and can be further adapted to settings with missing-not-at-random (MNAR) mechanisms by explicitly modeling the missingness process.

#### 4.2.2 | Unobserved Confounders and Modular StoNet

Many causal inference methods are built on the assumption that all relevant confounders are observed. In observational studies, however, this assumption is often difficult to justify. Proxy variables can provide partial information about latent confounders, especially in high-dimensional biomedical, social, and environmental applications. To formalize this setting, consider the causal structure shown in Figure 3a, which involves unobserved confounders Z, proxy variables X, treatment A, and outcome Y:

(18) $$X=f_{1}\left(Z,e_{x}\right),\;A=f_{2}\left(Z,e_{a}\right),\;Y=f_{3}\left(Z,A,e_{y}\right).$$

Motivated by the following factorization of the conditional distribution of the latent confounders,

(19) π(Z∣A,X,Y)∝π(Z)π(X∣Z)π(A∣Z)π(Y∣Z,A)∝π(Z∣X)π(A∣Z)π(Y∣Z,A),

Fang and Liang (2026) proposed modeling the causal DAG using a modular StoNet, as illustrated in Figure 3b. The resulting parameterization is given by

(20) $$\pi (Z|A,X,Y,\theta )\propto \pi \left(Z|X,\theta_{1}\right)\pi \left(A|Z,\theta_{2}\right)\pi \left(Y|Z,A,\theta_{3}\right),$$

where $\theta =\left(\theta_{1},\theta_{2},\theta_{3}\right)$, and the three factors are represented by modular networks parameterized by $\theta_{1},\theta_{2}$, and $\theta_{3}$, respectively. The modular StoNet can then be trained using an adaptive SG-MCMC algorithm, such as Algorithm 1.

Under the assumed causal DAG and standard causal-effect identification conditions, the do-calculus (Pearl 2009) gives

(21) $$\pi \left(Y|X,\text{do}\left(A=a\right)\right)=\int \pi \left(Y|Z,a\right)\pi \left(Z|X\right)dZ.$$

This identity suggests estimating the counterfactual outcome function by

$${\hat{Y}}_{X}(a)=\frac{1}{M}\sum_{m=1}^{M}\mu_{3}\left(Z^{\left(m\right)},a;{\overset{ˆ}{\theta }}_{3}\right),$$

where $Z^{\left(1\right)},\ldots ,Z^{\left(M\right)}$ are random samples drawn from $\pi \left(Z|X;{\overset{ˆ}{\theta }}_{1}\right)$. The individual and average treatment effect estimates can then be obtained by comparing ${\hat{Y}}_{X}\left(a_{1}\right)$ and ${\hat{Y}}_{X}\left(a_{0}\right)$. Fang and Liang (2026) established the consistency of these estimators under suitable regularity and sparsity conditions, although the latent variable Z is only identifiable up to loss-invariant transformations.

Fang and Liang (2026) evaluated the method using a dataset of twin births in the United States from 1989 to 1991. The treatment variable is binary, with A=1 denoting the heavier twin at birth, and the outcome variable is also binary, with Y=1 indicating mortality within the first year. Because both twins in each pair are observed, the twin-pair records can be viewed as paired potential outcomes, making it possible to compute the true ATE for evaluation. One important covariate in the dataset is *gestat10*, which records gestational age in weeks before birth. In their study, *gestat10* is treated as an unobserved confounder and imputed from proxy information during the training of the modular StoNet. Their numerical results show that this unobserved-confounder formulation improves treatment effect estimation relative to benchmark methods that do not explicitly model the unobserved confounder, as well as relative to some existing deep latent-variable models, such as the causal effect variational autoencoder (CEVAE) (Louizos et al. 2017).

We note that the modular StoNet provides a general framework for handling unobserved confounders in causal inference. Given a causal DAG, one can construct a modular StoNet by replacing each unknown functional relationship among the nodes with a DNN, and then train the resulting modular StoNet using an adaptive SG-MCMC algorithm. Instead of using the parameterization in (20), one may also construct the modular StoNet directly from the factorization given in the first line of (19). In this case, the direction of the arrows between X and Z in Figure 3 can be reversed, and the distribution of Z can be more freely specified. The modular StoNet also addresses several limitations of existing approaches for unobserved-confounder problems. In particular, it preserves the randomness of the unobserved confounder.

#### 4.2.3 | Granger Causality

Granger causality (Granger 1969) provides a classical framework for inferring directed predictive dependence in multivariate time series, which arise frequently in areas such as finance and neuroscience. In this framework, a process X is said to *Granger-cause* another process Y if the past history of X improves the prediction of Y beyond what can be achieved from the past history of Y alone. In multivariate settings, this notion is typically defined after conditioning on the histories of the remaining observed processes. The identification and estimation of Granger-causal relationships have been extensively studied in the literature; see, for example, Sims (1980), Marinazzo et al. (2008), Diks and Wolski (2016), Siggiridou and Kugiumtzis (2021), Tank et al. (2022), Zhou et al. (2024).

To enable Granger-causality inference for high-dimensional nonlinear time series, Ahn et al. (2026) proposed a deep state-space model. Let $\left\{y_{1},y_{2},\ldots ,y_{T}\right\}$ denote a stationary p-variate time series, where $y_{t}={\left(y_{t}^{\left(1\right)},\ldots ,y_{t}^{\left(p\right)}\right)}^{⊤}\in R^{p}$ for t=1,2,…,T. Equivalently, the data can be viewed as p coupled temporal sequences observed over T time points. The process is modeled through the following StoNet-based state-space model:

(22) $$x_{t}^{\left(i\right)}=g\left(x_{t-1}^{\left(i\right)},y_{t-1}^{\left(i\right)};w\right)+\epsilon_{t}^{\left(i\right)},\;i=1,2,\ldots ,p,\;y_{t}=\beta_{0}+Bx_{t}+e_{t},$$

where $x_{t}^{\left(i\right)}$ denotes the latent state of the ith series at time $t,\beta_{0}\in R^{p}$ is an intercept vector, $B\in R^{p\times p}$ is a loading matrix, and g(·) is a neural network parameterized by w. The innovations satisfy

$$\epsilon_{t}^{\left(i\right)}\overset{\text{iid}}{\sim }N\left(0,\tau^{2}\right),\;e_{t}={\left(e_{t}^{\left(1\right)},\ldots ,e_{t}^{\left(p\right)}\right)}^{⊤}\overset{\text{iid}}{\sim }N_{p}\left(0,\sigma^{2}I_{p}\right).$$

By construction, each latent coordinate $x_{t}^{\left(i\right)}$ depends only on the past observation $y_{t-1}^{\left(i\right)}$ of its corresponding series and its own past state $x_{t-1}^{\left(i\right)}$. Thus, within-series nonlinear dynamics are summarized locally in $x_{t}={\left(x_{t}^{\left(1\right)},\ldots ,x_{t}^{\left(p\right)}\right)}^{⊤}$, whereas cross-series predictive influence is mediated entirely through the loading matrix B. In particular, a nonzero entry $\beta_{ij}=[B{]}_{ij}$ indicates that $x_{t}^{\left(j\right)}$ contributes to the prediction of $y_{t}^{\left(i\right)}$. Under the model assumptions, a nonzero entry $\beta_{ij}$ is therefore interpreted as evidence that the jth series Granger-causes the ith series.

The model (22) can be viewed as a modular StoNet, with the latent states $x_{t}$ forming the stochastic layer. It can therefore be trained using an adaptive SG-MCMC algorithm. In particular, Ahn et al. (2026) developed an adaptive SGLD algorithm under the temporal setting, motivated by the adaptive LEnKF algorithm (Dong et al. 2023). The adaptive SGLD algorithm alternates between sampling the latent states and updating the parameters $\left\{w,\beta_{0},B,\tau^{2},\sigma^{2}\right\}$. To ensure consistent identification of the Granger-causality structure under this deep state-space model, Ahn et al. (2026) further imposed the mixture Gaussian prior (Sun et al. 2022a) on both w and B.

### 4.3 | Nonlinear Variable Selection

Sparse deep learning has emerged as a principled framework for nonlinear variable selection in complex systems; see, for example, Liang et al. (2018), Sun et al. (2022a), Sun et al. (2022b), and Zhang et al. (2023). As a further development in this direction, Sun and Liang (2027) proposed training a sparse StoNet with all modules being linear and with a Lasso penalty (Tibshirani 1996) imposed on the parameters θ. More precisely, Sun and Liang (2027) proposed fitting the StoNet in (1) by minimizing a penalized negative log-likelihood of the form

(23) $${\hat{\theta }}_{n}=\text{arg}\;\underset{\theta \in \Theta }{\text{min}}\left\{-\frac{1}{n}\sum_{i=1}^{n}\text{log}\;\pi \left(Y^{\left(i\right)},Y_{\text{mis}}^{\left(i\right)}|X^{\left(i\right)},\theta \right)+P_{\lambda_{n}}(\theta )\right\},$$

where $P_{\lambda_{n}}(\theta )$ denotes the Lasso penalty with regularization parameter $\lambda_{n}$. Using the factorization in (15), the optimization problem in (23) can be decomposed into a sequence of Lasso-penalized generalized linear model (GLM) subproblems. This decomposition leads naturally to Algorithm 2.

Sun and Liang (2027) studied the consistency of ${\hat{\theta }}_{n}$ in the simplified setting where the output layer consists of a single neuron. Under this setting, existing sparse-learning results for high-dimensional linear regression (Zhao and Yu 2006) and high-dimensional logistic regression (Huang et al. 2008) can be transferred directly to StoNet.

**Theorem 3. (Theorem 1 and Corollary 1 of** Sun and Liang 2027**).** *Suppose that all modules in the StoNet* (1) *are linear, a Lasso penalty is imposed on* θ, *and that Assumptions 1–2, together with suitable sparsity, sparse-eigenvalue, penalty-scaling, and local-contraction conditions, hold. Then, for all sufficiently large* n *and sufficiently large* t, *the IRO estimator satisfies*

$$\left\|{\overset{ˆ}{\theta }}_{n}^{\left(t\right)}-\theta^{*}\right\|\overset{p}{\to }0,$$

*and*

$$P\left({\hat{\gamma }}_{n}^{\left(t\right)}=\gamma^{*}\right)\to 1,$$

*where* $\theta^{*}$ *denotes the true parameter vector of the StoNet,* $\gamma^{*}$ *denotes its true sparse structure, and* ${\overset{ˆ}{\gamma }}_{n}^{\left(t\right)}$ *denotes the structure estimate obtained at iteration* t *of* Algorithm 2.

Sun and Liang (2027) provided a constructive proof of Theorem 3 by combining the convergence theory of the IRO algorithm (Liang, Jia, et al. 2018) with sparse-learning theory for Lasso regression (Zhao and Yu 2006) and Lasso classification (Huang et al. 2008). Extensions of their framework to other amenable penalties (Loh and Wainwright 2017), such as SCAD (Fan and Li 2001) and MCP (Zhang 2010), are straightforward. Moreover, because StoNet and the corresponding DNN are asymptotically equivalent, the consistency guarantees established in Theorem 3 extend to DNNs as well. In this sense, StoNet provides a bridge for transferring sparse-learning methodology and theory from linear models to DNNs.

Algorithm 2 facilitates the proof of parameter estimation consistency and structure-selection consistency for sparse StoNets. However, it does not scale well to massive datasets since each iteration requires full-data evaluation. To improve computational efficiency, Sun and Liang (2027) instead trained sparse StoNets using an adaptive SG-MCMC algorithm. Both the IRO and adaptive SG-MCMC algorithms aim to solve the same penalized optimization problem in (23).

### 4.4 | Prediction Intervals of Sparse StoNets

Consider a StoNet with all modules being linear. Its layered Markov structure provides a natural way to propagate and quantify latent-variable uncertainty across layers through repeated use of Eve’s law, also known as the law of total variance. To make this explicit, consider a regression StoNet fitted by Algorithm 2. Let z be a test input at which predictive uncertainty is to be assessed. For layer ℓ and IRO iteration t, let $Z_{\ell }^{\left(t\right)}$ denote the imputed latent vector corresponding to input z. We set $Z_{0}^{\left(t\right)}=z$ for all t. Then, for any layer ℓ∈{2,…,h+1}, Eve’s law yields

(25) $${𝚺}_{\ell }^{\left(t\right)}=E\left\{\text{Var}\left(Z_{\ell }^{\left(t\right)}|Z_{\ell -1}^{\left(t\right)}\right)\right\}+\text{Var}\left\{E\left(Z_{\ell }^{\left(t\right)}|Z_{\ell -1}^{\left(t\right)}\right)\right\}.$$

**ALGORITHM 3 |:** Prediction Interval Construction for StoNet (Sun and Liang 2027).

1: **Input:** A collection of StoNet parameter estimates $𝒮=\left\{{\overset{ˆ}{\theta }}_{n}^{\left(1\right)},{\overset{ˆ}{\theta }}_{n}^{\left(2\right)},\ldots ,{\overset{ˆ}{\theta }}_{n}^{\left(m\right)}\right\}$, and a test point z.
2: **for** t=1,2,…,m **do**
3: **for** ℓ=1,2,…,h+1 **do**
4: Compute the estimated latent-variable covariance matrix ${\hat{𝚺}}_{\ell }^{\left(t\right)}$ based on (25).
5: **end for**
6: **for** $j=1,2,\ldots ,d_{h+1}$ **do**
7: **Training error:** Estimate the residual variance by
$${\hat{\varsigma }}_{h+1,j}^{2(t)}=\frac{1}{n}\overset{n}{\underset{k=1}{\sum }}{\left\{y_{j}^{\left(k\right)}-\mu_{j}\left(x^{\left(k\right)},{\overset{ˆ}{\theta }}_{n}^{\left(t\right)}\right)\right\}}^{2},$$
where $y_{j}^{\left(k\right)}$ is the jth component of $y^{\left(k\right)}$, and $\left(y^{\left(k\right)},x^{\left(k\right)}\right)$ denotes the kth training observation.
8: **Prediction interval:** Form the 95% prediction interval
(26) $$\left(\mu_{j}\left(z,{\hat{\theta }}_{n}^{\left(t\right)}\right)-1.96\sqrt{{\hat{𝚺}}_{h+1,j}^{\left(t\right)}+{\overset{ˆ}{\varsigma }}_{h+1,j}^{2(t)}},\mu_{j}\left(z,{\hat{\theta }}_{n}^{\left(t\right)}\right)+1.96\sqrt{{\hat{𝚺}}_{h+1,j}^{\left(t\right)}+{\overset{ˆ}{\varsigma }}_{h+1,j}^{2(t)}}\right),$$
where ${\hat{𝚺}}_{h+1,j}^{\left(t\right)}$ denotes the (j,j) entry of ${\hat{𝚺}}_{h+1}^{\left(t\right)}$.
9: **end for**
10: **end for**
11: **Output:** For each $j\in \left\{1,2,\ldots ,d_{h+1}\right\}$, report the final 95% prediction interval for $Y_{j}(z)$ by averaging the corresponding lower and upper endpoints over t=1,…,m.

Let $\mu \left(z,{\overset{ˆ}{\theta }}_{n}\right)$ denote the StoNet prediction at z based on the parameter estimate ${\hat{\theta }}_{n}$. As in the usual forward pass, the injected latent noise is set to zero, so that the resulting prediction coincides with that of the corresponding DNN. Suppose that, after convergence, we retain a collection of StoNet parameter estimates $𝒮=\left\{{\overset{ˆ}{\theta }}_{n}^{\left(1\right)},\ldots ,{\overset{ˆ}{\theta }}_{n}^{\left(m\right)}\right\}$. Using a Wald-type approximation, a 95% prediction interval for the future response component $Y_{j}(z)$ can be constructed according to Algorithm 3; see Sun and Liang (2027) for details.

The asymptotic validity of the prediction intervals in (26) follows from the consistency of ${\overset{ˆ}{\theta }}_{n}^{\left(t\right)}$, the asymptotic normality of the post-selection layer-wise estimators, and the consistency of the corresponding variance estimators used in the covariance recursion.

### 4.5 | Uncertainty Quantification for Large-Scale DNNs

Large-scale DNNs can achieve excellent predictive accuracy but are often poorly calibrated (Guo et al. 2017). A useful two-stage strategy for uncertainty quantification is to first treat a pretrained DNN as a feature extractor and then fit a sparse head model to the resulting representations on validation data. The data efficiency of this strategy can be justified based on the asymptotic SDR property of learned representations (Liang et al. 2022).

In Sun and Liang (2027), the sparse head is specified as a sparse StoNet. Once fitted, this sparse StoNet head is attached to the pretrained feature extractor to provide calibrated predictive uncertainty. Empirical results on image-recognition datasets, such as CIFAR100, show that the resulting post-StoNet approach improves uncertainty calibration while preserving high predictive accuracy.

## 5 | Kernel-Expanded StoNets

The Gaussian assumption for the latent noises in (1) is not essential. Sun and Liang (2022) introduced the kernel-expanded StoNet (K-StoNet), in which the first-layer noise is modeled by an ε-insensitive Laplace distribution and other modules are linear. The associated conventional target model is a kernel-expanded neural network:

(27) $${\tilde{Y}}_{1}=b_{1}+\beta \phi (X),\;{\tilde{Y}}_{l}=b_{l}+w_{l}\Psi \left({\tilde{Y}}_{l-1}\right),\;l=2,\ldots ,h,\;Y=b_{h+1}+w_{h+1}\Psi \left({\tilde{Y}}_{h}\right)+e_{h+1},$$

where ϕ(X) denotes a kernel feature map. K-StoNet thus incorporates the kernel trick into deep learning while preserving the tractable layer-wise structure of StoNet.

Under assumptions similar to those used for StoNet, Sun and Liang (2022) showed that K-StoNet is asymptotically equivalent to the corresponding kernel-expanded neural network.

**ALGORITHM 4 |:** IRO Algorithm for K-StoNet Training (Sun and Liang 2022).

1: **Input:** Dataset $D_{n}=(Y,X)$, total number of iterations T, and the number of Monte Carlo steps $t_{\text{MC}}$.
2: **Initialization:** Randomly initialize the network parameters ${\overset{ˇ}{\theta }}^{\left(0\right)}=\left({\overset{ˇ}{\theta }}_{1}^{\left(0\right)},\ldots ,{\overset{ˇ}{\theta }}_{h+1}^{\left(0\right)}\right)$.
3: **for** t=1,2,…,T **do**
4: **(i) Imputation:** For each observation $\left(X^{\left(i\right)},Y^{\left(i\right)}\right)$, sample $Y_{\text{mis}}^{\left(i,t\right)}$ from
$$\pi \left(Y_{\text{mis}}^{\left(i\right)}|Y^{\left(i\right)},X^{\left(i\right)},{\overset{ˇ}{\theta }}^{\left(t-1\right)},\sigma_{n}^{2}\right)$$
using a Langevin-dynamics transition kernel iterated for $t_{\text{MC}}$ steps.
5: **(ii) Regularized optimization:** Based on the pseudo-complete data $\left\{\left(Y^{\left(i\right)},Y_{\text{mis}}^{\left(i,t\right)},X^{\left(i\right)}\right):i=1,\ldots ,n\right\}$, update the parameters layer by layer.
6: **(ii-1) SVR layer:** Compute
(28) $${\overset{ˇ}{\theta }}_{1}^{\left(t\right)}=\text{arg}\;\underset{\beta ,b_{1}}{\text{min}}\left\{\frac{{\tilde{C}}_{n,1}}{n}\sum_{i=1}^{n}{\left\|{\left|Y_{1}^{\left(i,t\right)}-\beta^{T}\phi \left(Y_{0}^{\left(i,t\right)}\right)-b_{1}\right|}_{\varepsilon }\right\|}_{1}+\frac{1}{2}\|\beta {\|}_{2}^{2}\right\},$$
where ${\tilde{C}}_{n,1}$ is the regularization parameter for the SVR layer.
7: **(ii-2) Regression layers:**
8: **for** l=2,3,…,h+1 **do**
9: Compute
(29) $${\overset{⌣}{\theta }}_{l}^{\left(t\right)}=\text{arg}\;\underset{\theta_{l}}{\text{min}}\left\{-\frac{1}{n}\sum_{i=1}^{n}\text{log}\;\pi \left(Y_{l}^{\left(i,t\right)}|Y_{l-1}^{\left(i,t\right)},\theta_{l},\sigma_{l}^{2}\right)+P_{\lambda_{n}}\left(\theta_{l}\right)\right\},$$
by solving a multivariate linear or logistic regression problem, where h is the number of hidden layers, $Y_{h+1}^{\left(i,t\right)}=Y^{\left(i\right)},Y_{0}^{\left(i,t\right)}=X^{\left(i\right)}$, and $\theta_{l}=\left\{W_{l},b_{l}\right\}$ denotes the parameters of layer l.
10: **end for**
11: **end for**
12: **Output:** The final estimate ${\overset{ˇ}{\theta }}^{\left(T\right)}=\left({\overset{ˇ}{\theta }}_{1}^{\left(T\right)},\ldots ,{\overset{ˇ}{\theta }}_{h+1}^{\left(T\right)}\right)$.

The main computational advantage of K-StoNet is that, conditional on the imputed latent variables, the first-layer parameters can be updated by solving a support vector regression (SVR) problem, whereas the remaining layers reduce to standard linear or logistic regression problems. Consequently, K-StoNet can be trained by solving a sequence of convex optimization problems, as described in Algorithm 4. More importantly, Sun and Liang (2022) showed that, when the hidden layers are arranged in a pyramid-shaped architecture, K-StoNet is immune to local traps: every local minimum of its energy landscape corresponds to a global optimum. This property is a consequence of the kernel expansion in the first hidden layer.

The numerical experiments in Sun and Liang (2022) show that K-StoNet can converge rapidly, often within only a small number of epochs. Their results also suggest that K-StoNet is empirically less susceptible to overfitting and tends to outperform conventional DNNs in prediction.

A recent development closely related to K-StoNet and StoNet is Wahkon, a deep RKHS superposition network introduced by Chen et al. (2026). Wahkon replaces the fixed activation functions of conventional neural networks with learned univariate link functions defined on the edges of the network, with each link function constrained to lie in a reproducing kernel Hilbert space. Its penalized estimator admits a finite-dimensional deep representer theorem and can be interpreted as a maximum a posteriori estimator under a hierarchical Gaussian-process prior. Wahkon and StoNet share the broad objective of making deep learning more statistically tractable through layerwise modeling, but they introduce probabilistic structure in different ways. StoNet adds explicit noise to selected hidden layers and treats the resulting hidden-layer outputs as latent variables, whereas Wahkon places Gaussianprocess priors on the layerwise link functions; conditional on these functions, its forward map remains deterministic. K-StoNet is particularly relevant to this comparison because it combines kernel methods with the latent-variable formulation of StoNet. However, K-StoNet introduces a kernel feature expansion in the first layer while retaining stochastic hidden representations, whereas Wahkon learns RKHS link functions throughout the network. These complementary perspectives suggest several directions for future research. For example, the adaptive SG-MCMC and uncertainty-propagation methods developed for StoNet may provide useful ideas for posterior computation in Wahkon, while Wahkon’s representer-theorem perspective and layerwise RKHS regularization may motivate further extensions of K-StoNet.

## 6 | Sublinearly Structured DNNs

Rather than treating StoNet as the primary object of learning, this section shows that StoNet can also be used as an analytical device, or auxiliary model, for studying properties of conventional DNNs by leveraging the asymptotic equivalence between the StoNet and DNN. In particular, Kim et al. (2026) showed that DNNs with sublinear structure can achieve feature-learning consistency, even though they remain over-parameterized in the usual sense that the number of parameters may exceed the training sample size. This differs from sparse deep learning theory, where consistency is typically established under the condition that the total number of effective parameters is o(n) (Liang, Li, and Zhou 2018; Farrell et al. 2021; Sun et al. 2022a). The analysis relies on the following two key notions.

**Definition 1.** [Kim et al. 2026] A DNN is said to be a sublinearly structured DNN, or simply a sublinear DNN, if its architecture satisfiesh

(30) $$d_{0}≺n,\;d_{h+1}≺n,\;\sum_{l=1}^{h}d_{l}≺n,$$

where $d_{0}$ denotes the input dimension, $d_{l}$ is the width of hidden layer l,h is the number of hidden layers, $d_{h+1}$ is the output dimension, and n is the training sample size.

**Definition 2.** [Radhakrishnan et al. 2024] A neural feature is defined as an eigenvector of $w_{l}^{⊤}w_{l}$, where $w_{l}\in R^{d_{l}\times d_{l-1}}$ is the weight matrix at hidden layer l, and $d_{l}$ is the width of that layer.

Definition 2 is adopted from Radhakrishnan et al. (2024). Under this definition, consistency in estimating $w_{l}$, up to loss-invariant transformations, can be interpreted as *feature-learning* consistency.

### 6.1 | Feature Learning Consistency

Consider again the StoNet model in (1). Let ${\bar{w}}_{l}=\left(b_{l},w_{l}\right)\in R^{d_{l}\times \left(d_{l-1}+1\right)}$ denote the augmented parameter matrix of layer l, including the bias term, and write $\theta =\left({\bar{w}}_{1},\ldots ,{\bar{w}}_{h+1}\right)$. Define the $\ell_{2}$-aggregated layerwise operator-norm distance by

(31) $$d_{\text{op}}\left(\theta ,\theta^{\prime }\right)≔{\left\|\theta -\theta^{\prime }\right\|}_{d_{\text{op}}}≔{\left\{\sum_{l=1}^{h+1}{\left\|{\bar{w}}_{l}-{\bar{w}}_{l}^{\prime }\right\|}_{\text{op}}^{2}\right\}}^{1/2},$$

where $\|A{\|}_{\text{op}}=\sqrt{\lambda_{\text{max}}\left(A^{⊤}A\right)}$. In addition to the sublinear architecture condition, Kim et al. (2026) assume that the hidden-layer noise levels are chosen so that

(32) $$a_{n}^{2}≔\frac{1}{n}\sum_{l=1}^{h+1}\left(d_{l}+d_{l-1}+1\right)\frac{\sigma_{l}^{2}}{\sigma_{l-1}^{2}}\to 0,\;n\to \infty .$$

Here $\sigma_{0}^{2}=\kappa_{\text{min}}$ is fixed, $\sigma_{h+1}=O(1)$, and the hidden-layer noise levels can be chosen in increasing order, $\sigma_{1}<\sigma_{2}<\cdots <\sigma_{h}<\sigma_{h+1}$.

Corresponding to the operator-norm metric, Assumption 2 can be updated as follows:

**Assumption 4.** (i) The expected loss function $Q^{*}(\theta )$, defined in (5), is continuous in θ and uniquely maximized at $\theta^{*}$ (up to loss-invariant transformations); (ii) for any ϵ>0, ${\text{sup}}_{\theta \in \Theta \setminus B(\epsilon )}Q^{*}(\theta )$ exists, where $B(\epsilon )=\left\{\theta :d_{\text{op}}\left(\theta ,\theta^{*}\right)<\epsilon \right\}$, and $\delta_{\epsilon }=Q^{*}\left(\theta^{*}\right)-{\text{sup}}_{\theta \in \Theta \setminus B\left(\epsilon \right)}Q^{*}(\theta )>0$.

Within the IRO framework (Liang, Jia, et al. 2018), Kim et al. (2026) established that Algorithm 2 produces consistent feature learning for sublinearly structured DNNs. In this setting, they set the penalty $P_{\lambda_{n}}(\theta )=0$, because the architectural constraint (30) already provides sufficient control of model complexity. Let ${\overset{ˆ}{\theta }}_{n}^{\left(t\right)}$ denote the IRO estimate of $\theta^{*}$ obtained at iteration t, where both ${\overset{ˆ}{\theta }}_{n}^{\left(t\right)}$ and $\theta^{*}$ are understood up to loss-invariant transformations.

**Theorem 4. (Lemma 6 and Theorem 1 of** Kim et al. 2026**).** *Suppose Assumptions 1 and 4, condition* (32), *and the required regularity conditions hold. If the network architecture satisfies the sublinear scaling condition in* (30), *then, for every* ϵ>0,

$$P\left\{d_{op}\left({\overset{ˆ}{\theta }}_{n}^{\left(t\right)},\theta^{*}\right)>\epsilon \right\}\to 0,\;\text{as}\;n\to \infty \;\text{and}\;t\to \infty .$$

*In particular, for each hidden layer* l=1,…,h, *define*

$$A_{l}^{*}=w_{l}^{*⊤}w_{l}^{*},\;{\hat{A}}_{l}^{\left(t\right)}={\hat{w}}_{l}^{(t)⊤}{\hat{w}}_{l}^{\left(t\right)},$$

*where* $w_{l}^{*}$ *and* $w_{l}^{*}$ *are corresponding weight matrices in* $\theta^{*}$ *and* ${\overset{ˆ}{\theta }}_{n}^{\left(t\right)}$, respectively. Let $\lambda_{1}\left(A_{l}^{*}\right)\geq \cdots \geq \lambda_{d_{l-1}}\left(A_{l}^{*}\right)$ and $\lambda_{1}\left({\overset{ˆ}{A}}_{l}^{\left(t\right)}\right)\geq \cdots \geq \lambda_{d_{l-1}}\left({\overset{ˆ}{A}}_{l}^{\left(t\right)}\right)$ *denote their ordered eigenvalues. Then*

$$\underset{1\leq j\leq d_{l-1}}{max}\left|\lambda_{j}\left({\hat{A}}_{l}^{\left(t\right)}\right)-\lambda_{j}\left(A_{l}^{*}\right)\right|\overset{p}{\to }0,\;\text{as}\;n\to \infty \;\text{and}\;t\to \infty .$$

*Moreover, let* $V_{l}^{*}$ *and* ${\overset{ˆ}{V}}_{l}^{\left(t\right)}$ *contain orthonormal bases for the top* $r_{l}$-*dimensional eigenspaces of* $A_{l}^{*}$ *and* ${\hat{A}}_{l}^{\left(t\right)}$, respectively. If the eigengap condition $\lambda_{r_{l}}\left(A_{l}^{*}\right)-\lambda_{r_{l}+1}\left(A_{l}^{*}\right)\geq \delta_{l}>0$ holds for some constant $\delta_{l}$, *then*

$${\left\|{\hat{V}}_{l}^{\left(t\right)}{\hat{V}}_{l}^{(t)⊤}-V_{l}^{*}V_{l}^{*⊤}\right\|}_{op}\overset{p}{\to }0,\;\text{as}\;n\to \infty \;\text{and}\;t\to \infty .$$

*Thus, the DNN estimator is consistent for both the eigenvalues and the corresponding feature subspaces.*

By Theorem 4, sublinear DNNs can achieve eigenvalue and feature-learning consistency, even though they may be over-parameterized in the conventional parameter-count sense. However, eigenvalue and feature-learning consistency alone do not imply prediction consistency. Prediction consistency also requires control of the forward-stability factor, which measures how layerwise operator-norm errors propagate and are amplified through the forward pass of the neural network. Kim et al. (2026) further show that a sublinear DNN can achieve prediction consistency when the learned representations have bounded effective dimension and the depth remains fixed as n grows.

### 6.2 | Approximation Power

Theorem 4 implicitly requires that the sublinear DNN possess sufficient approximation power for the target function. Under the architectural constraint in (30), Kim et al. (2026) investigated the approximation ability of sublinear DNNs for hierarchically compositional function classes, motivated by the inherently compositional structure of deep networks. Based on the approximation results of Poggio et al. (2017), they established the following result.

**Theorem 5. (Theorem 3 of** Kim et al. 2026**).** *Let* f(x) *be defined on a compact subset of* $R^{d_{0}}$, *and suppose that* f *admits a hierarchical compositional representation in which each constituent function depends on at most* s *variables, where* $s\leq d_{0}$ *is fixed*.

- *If* f *is Lipschitz continuous and $d_{0}=O\left(n^{\alpha }\right)$ for some* 0<α<1, *then, for any* $\varepsilon =n^{-(1-\alpha -\delta )/s}$ *with* 0<δ<1-α, *there exists a sublinear ReLU DNN such that* $\left\|f-f_{\theta }\right\|\leq \varepsilon$ *as* n→∞, *where* $f_{\theta }$ *denotes the function represented by the network*.
- *If* f *is continuously differentiable and* $d_{0}=O\left(n^{\alpha }\right)$ *for some* 0<α<1, *then the same approximation conclusion holds for sublinear DNNs with smooth activation functions, such as sigmoid or tanh*.

Kim et al. (2026) also conducted an architectural analysis of standard CNNs, including AlexNet, VGG, ResNet, and GoogLeNet. Their analysis shows that these widely used large-scale CNN architectures satisfy the sublinear scaling conditions when trained on benchmark datasets such as ImageNet, CIFAR10, CIFAR100, and MNIST, each of which contains at least n≥50,000 training samples. Although these networks may contain a large number of parameters, both the maximum incoming trainable dimension per unit and the total number of trainable units remain controlled relative to the sample size.

Since image data naturally exhibit hierarchical and compositional structure, Theorems 4 and 5 provide useful theoretical support for understanding the success of these architectures. Together, these theoretical results and empirical architectural summaries offer a principled explanation for why large-scale CNNs can achieve excellent predictive accuracy when trained on sufficiently large datasets.

## 7 | Conclusion

This article provides a concise review of StoNet, covering its formulation, theoretical foundations, computational methods, and applications. StoNet and its variants—including Causal-StoNet, K-StoNet, sparse StoNet heads, and sublinearly structured DNNs—demonstrate how probabilistic modeling and deep learning can be integrated to address fundamental inferential challenges in modern data science. Table 1 summarizes the main applications and extensions discussed in this article, together with their computational methods, available software implementations, and references.

The central message is that, when stochasticity is introduced through a coherent latent-variable structure, deep neural networks become more statistically analyzable: their internal structures are more interpretable, and their prediction uncertainty is more quantifiable. In this way, StoNet has broadened the scope of deep learning from prediction-oriented function approximation to a richer framework for statistical inference.

## Figures and Tables

**FIGURE 1 |:**
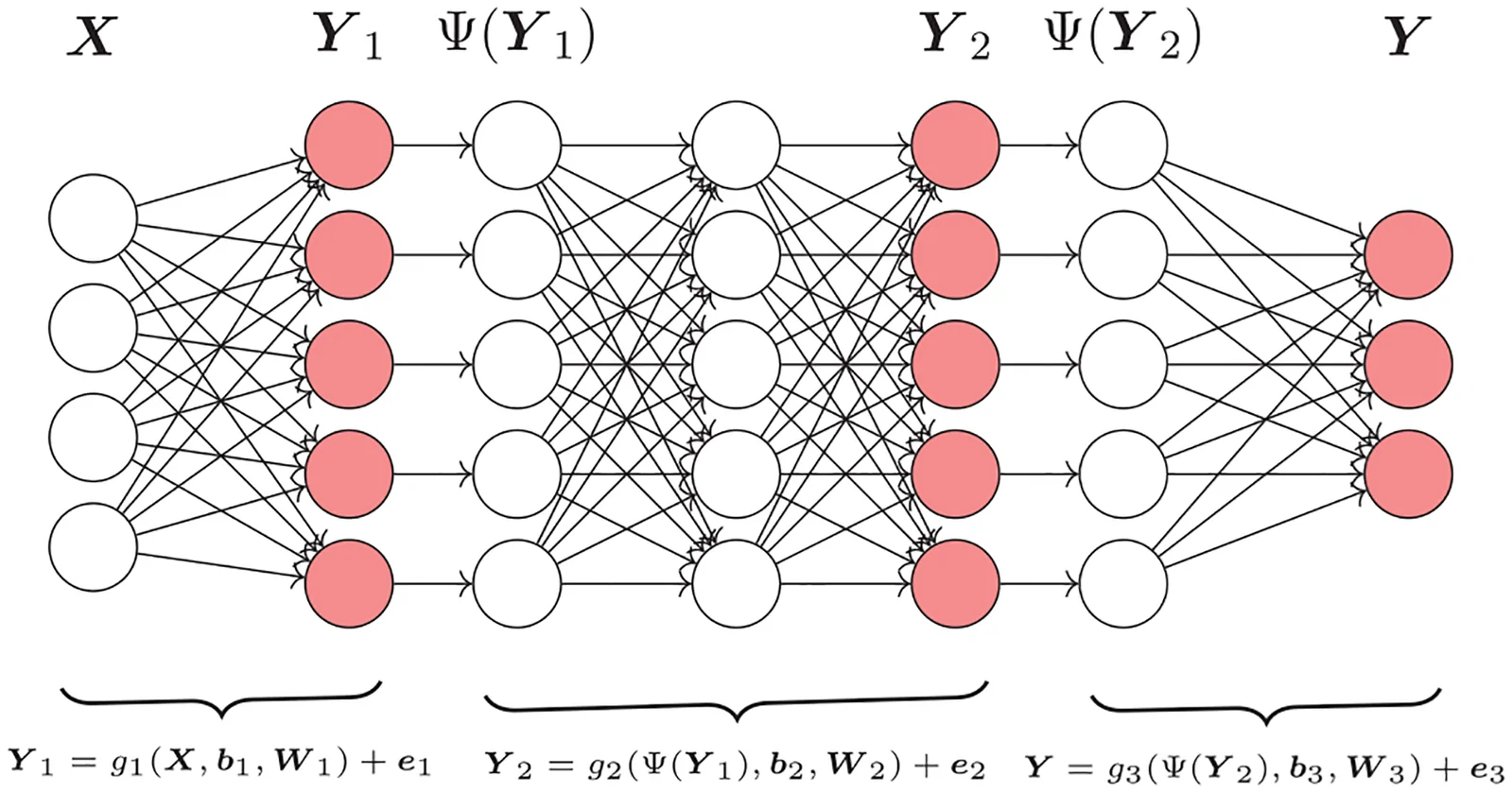
A schematic illustration of the StoNet architecture. The vector X denotes the input variables, while $Y_{1}=g_{1}\left(X,b_{1},W_{1}\right)+e_{1}$ and $Y_{2}=g_{2}\left(\Psi \left(Y_{1}\right),b_{2},W_{2}\right)+e_{2}$ are latent variables that feed into the corresponding hidden-layer units. The output variables are given by $Y=g_{3}\left(\Psi \left(Y_{2}\right),b_{3},W_{3}\right)+e_{3}$, and Ψ(·) denotes the element-wise activation function.

**FIGURE 2 |:**
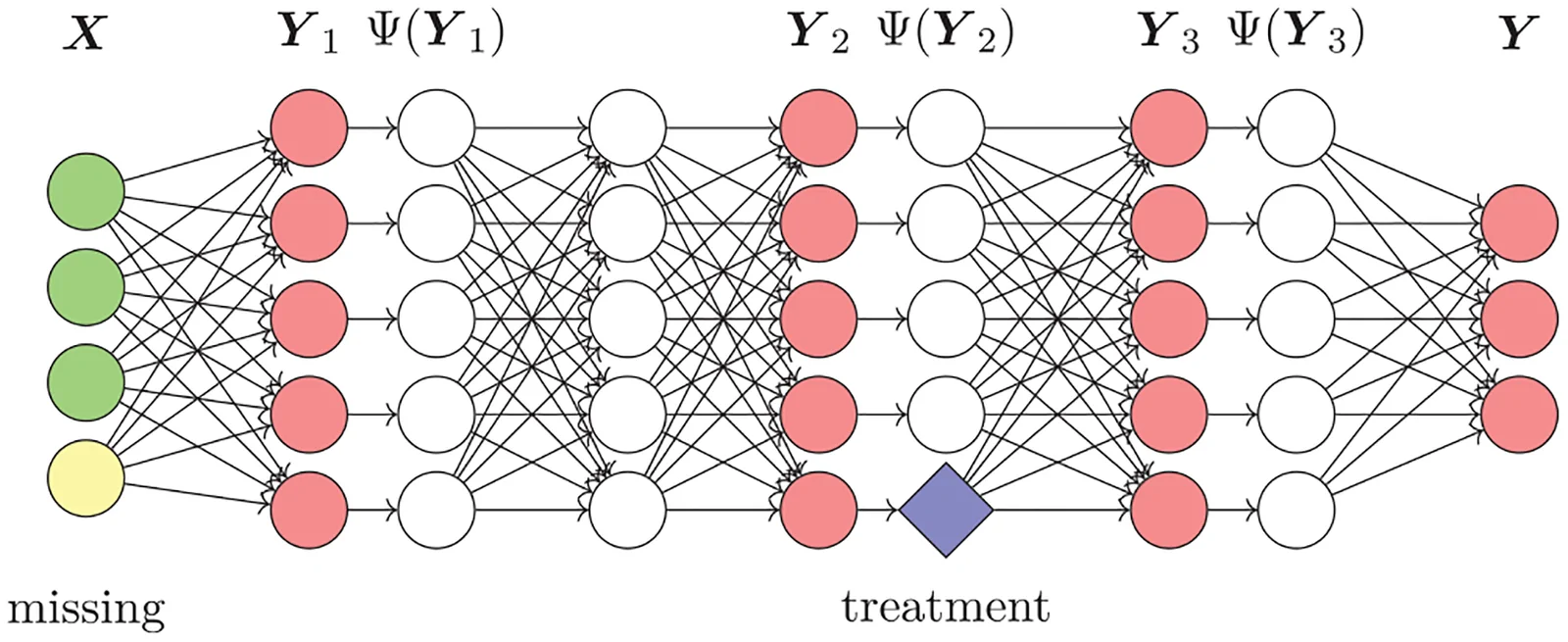
Illustration of the Causal-StoNet architecture. Nodes shaded in green correspond to observed variables, whereas those in yellow indicate missing values. The diamond-shaped node denotes the treatment variable, which is incorporated as a visible unit in an intermediate layer.

**FIGURE 3 |:**
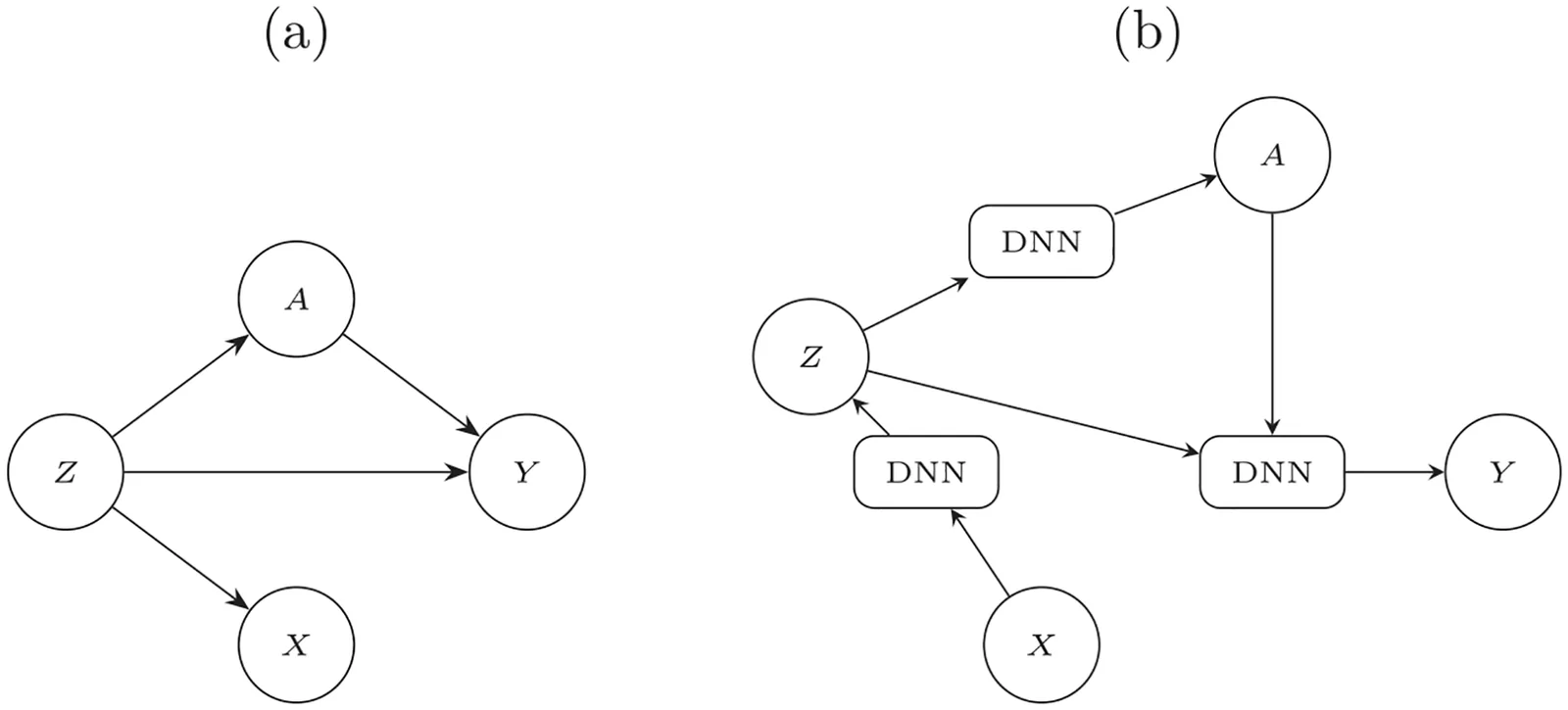
(a) Causal DAG, where X,Z,A, and Y denote proxies, unobserved confounders, treatment, and outcome, respectively; (b) architecture of modular StoNet with proxies.

**TABLE 1 |:** Summary of the main applications and extensions of StoNet.

Variants of StoNet	Statistical task	Computational method	References
StoNet^a^	Nonlinear sufficient dimension reduction	Adaptive SGHMC	Liang et al. (2022)
Causal-StoNet^b^	Causal inference with high-dimensional observed confounders	Adaptive SG-MCMC	Fang and Liang (2024)
CI-StoNet	Causal inference with unobserved confounders	Adaptive SGHMC	Fang and Liang (2026)
Deep state-space model	Granger-causality inference for high-dimensional nonlinear time series	Adaptive SGLD	Ahn et al. (2026)
Sparse StoNet	Nonlinear variable selection	Adaptive SG-MCMC with sparsity penalties	Sun and Liang (2027)
Post-StoNet	Uncertainty quantification for pretrained large-scale DNNs	Two-stage training with a pretrained feature extractor and a sparse StoNet head	Sun and Liang (2027)
K-StoNet^c^	Kernel-expanded deep learning, prediction, and uncertainty quantification	IRO with support vector regression and layerwise regression	Sun and Liang (2022)
Sublinearly structured DNN^d^	Feature-learning consistency and architecture design	IRO-based theoretical and numerical analysis	Kim et al. (2026)

*Note*: The software packages and code for selected StoNet applications are publicly available.

a https://github.com/booml247/StoNet_SDR_NeurIPS.

b https://github.com/nixay/Causal-StoNet.

c https://github.com/sylydya/A-Kernel-Expanded-Stochastic-Neural-Network.

d https://github.com/sehwankimstat/Sublinear-DNN.

## Data Availability

Data sharing not applicable to this article as no datasets were generated or analysed during the current study.

## Footnotes

1 For binary classification, let $\eta \left(Y_{h};b_{h+1},W_{h+1}\right)$ denote the output logit, and the temperature-scaled success probability is defined by

$$\text{Pr}\left(Y=1|Y_{h}\right)=\frac{\text{exp}\;\left\{\eta \left(Y_{h};b_{h+1},W_{h+1}\right)/\sigma_{h+1}^{2}\right\}}{1+\text{exp}\;\left\{\eta \left(Y_{h};b_{h+1},W_{h+1}\right)/\sigma_{h+1}^{2}\right\}}.$$

For multiclass classification, the corresponding softmax probabilities are defined by

$$\text{Pr}\left(Y=k|Y_{h}\right)=\frac{\text{exp}\;\left\{\eta_{k}\left(Y_{h};b_{h+1},W_{h+1}\right)/\sigma_{h+1}^{2}\right\}}{\sum_{j=1}^{K}\text{exp}\;\left\{\eta_{j}\left(Y_{h};b_{h+1},W_{h+1}\right)/\sigma_{h+1}^{2}\right\}},k=1,\ldots ,K.$$

Thus, $\sigma_{h+1}^{2}$ controls the sharpness of the class probabilities, playing a role analogous to the output-noise variance in Gaussian regression.

## References

[R1] Ackley DH, Hinton GE, and Sejnowski TJ. 1985. “A Learning Algorithm for Boltzmann Machines.” Cognitive Science 9: 147–169.

[R2] Ahn H, Song Q, and Liang F. 2026. “Granger Causality Inference for High-Dimensional Nonlinear Time Series With a Deep Particle Filter.” Manuscript Submitted.

[R3] Bura E, and Cook RD. 2001. “Estimating the Structural Dimension of Regressions via Parametric Inverse Regression.” Journal of the Royal Statistical Society, Series B 63: 393–410.

[R4] Celeux G, and Diebolt J. 1985. “The Sem Algorithm: A Probabilistic Teacher Algorithm Derived From the Em Algorithm for the Mixture Problem.” Computational Statistics Quarterly 2: 73–82.

[R5] Chen Y, Zhong W, and Ma P. 2026. “Wahkon: A Statistically Principled Deep RKHS Superposition Network.” arXiv preprint arXiv:2605.14041.

[R6] Cook RD. 2000. “Save: A Method for Dimension Reduction and Graphics in Regression.” Communications in Statistics - Theory and Methods 29, no. 9–10: 2109–2121.

[R7] Cook RD, and Weisberg S. 1991. “Discussion of ‘Sliced Inverse Regression for Dimension Reduction,’ by k.c. Li.” Journal of the American Statistical Association 86: 328–332.

[R8] Deng W, Zhang X, Liang F, and Lin G. 2019. “An Adaptive Empirical Bayesian Method for Sparse Deep Learning.” Advances in Neural Information Processing Systems 32: 5564–5574. PMC7687285 33244209

[R9] Diks C, and Wolski M. 2016. “Nonlinear Granger Causality: Guidelines for Multivariate Analysis.” Journal of Applied Econometrics 31, no. 7: 1333–1351.

[R10] Dong T, Zhang P, and Liang F. 2023. “A Stochastic Approximation-Langevinized Ensemble Kalman Filter for State Space Models With Unknown Parameters.” Journal of Computational and Graphical Statistics 32, no. 2: 448–469. 10.1080/10618600.2022.2107531 PMC10795543 38240013

[R11] Fan J, and Li R. 2001. “Variable Selection via Nonconcave Penalized Likelihood and Its Oracle Properties.” Journal of the American Statistical Association 96: 1348–1360.

[R12] Fang Y, and Liang F. 2024. “Causal-StoNet: Causal Inference for High-Dimensional Complex Data.” ICLR. PMC12918773 41725678

[R13] Fang Y, and Liang F. 2026. “Stochastic Neural Networks for Causal Inference With Missing Confounders.” ICLR. PMC13717250 42959562

[R14] Farrell M, Liang T, and Misra S. 2021. “Deep Neural Networks for Estimation and Inference.” Econometrica 89: 181–213.

[R15] Fertl L, and Bura E. 2022. “Conditional Variance Estimator for Sufficient Dimension Reduction.” Bernoulli 28, no. 3: 1862–1891.

[R16] Fukumizu K, Bach FR, and Jordan MI. 2009. “Kernel Dimension Reduction in Regression.” Annals of Statistics 37, no. 4: 1871–1905.

[R17] Fukumizu K, and Leng C. 2014. “Gradient-Based Kernel Dimension Reduction for Regression.” Journal of the American Statistical Association 109: 359–370.

[R18] Gal Y, and Ghahramani Z. 2016. “Dropout as a Bayesian Approximation: Representing Model Uncertainty in Deep Learning.” In Proceedings of the 33rd International Conference on International Conference on Machine Learning – Volume 48, 1050–1059. ICML.

[R19] Gao X, Gürbüzbalaban M, and Zhu L. 2021. “Global Convergence of Stochastic Gradient Hamiltonian Monte Carlo for Nonconvex Stochastic Optimization: Nonasymptotic Performance Bounds and Momentum-Based Acceleration.” Operations Research 70, no. 5: 2931–2947.

[R20] Granger CWJ. 1969. “Investigating Causal Relations by Econometric Models and Cross-Spectral Methods.” Econometrica 37, no. 3: 424–438.

[R21] Gülçehre C, Moczulski M, Denil M, and Bengio Y. 2016. “Noisy Activation Functions.” ICML.

[R22] Guo C, Pleiss G, Sun Y, and Weinberger KQ. 2017. “On Calibration of Modern Neural Networks.” In In International Conference on Machine Learning, 1321–1330. PMLR.

[R23] Hinton GE. 2002. “Training Products of Experts by Minimizing Contrastive Divergence.” Neural Computation 14: 1771–1800. 10.1162/089976602760128018 12180402

[R24] Hinton GE, Osindero S, and Teh YW. 2006. “A Fast Learning Algorithm for Deep Belief Nets.” Neural Computation 18: 1527–1554. 10.1162/neco.2006.18.7.1527 16764513

[R25] Hinton GE, and Salakhutdinov R. 2006. “Reducing the Dimensionality of Data With Neural Networks.” Science 313: 504–507. 10.1126/science.1127647 16873662

[R26] Hinton GE, Srivastava N, Krizhevsky A, Sutskever I, and Salakhutdinov RR. 2012. “Improving Neural Networks by Preventing Co-Adaptation of Feature Detectors.” arXiv Preprint arXiv:1207.0580.

[R27] Hristache M, Juditsky A, Polzehl J, and Spokoiny V. 2001. “Structure Adaptive Approach for Dimension Reduction.” Annals of Statistics 29, no. 6: 1537–1566.

[R28] Huang J, Ma S, and Zhang C-H. 2008. “The Iterated Lasso for High-Dimensional Logistic Regression.” The University of Iowa, Department of Statistics and Actuarial Sciences, Pages 1–20.

[R29] Imbens G. 2004. “Nonparametric Estimation of Average Treatment Effects Under Exogeneity: A Review.” Review of Economics and Statistics 86: 4–29.

[R30] Kim S, Sun Y, and Liang F. 2026. “Sublinearly Structured Deep Neural Networks Achieve Feature Learning Consistency for Compositional Functions.” Statistical Learning and Data Science 1: 100007.

[R31] Kingma DP, and Welling M. 2014. “Auto-Encoding Variational Bayes.” In International Conference on Learning Representations. ICLR.

[R32] Lee K-Y, Li B, and Chiaromonte F. 2013. “A General Theory for Nonlinear Sufficient Dimension Reduction: Formulation and Estimation.” Annals of Statistics 41, no. 1: 221–249.

[R33] Li B, and Wang S. 2007. “On Directional Regression for Dimension Reduction.” Journal of the American Statistical Association 102: 997–1008.

[R34] Li BD, Zha H, and Chiaromonte F. 2005. “Contour Regression: A General Approach to Dimension Reduction.” Annals of Statistics 33: 1580–1616.

[R35] Li K-C. 1991. “Sliced Inverse Regression for Dimension Reduction.” Journal of the American Statistical Association 86, no. 414: 316–327.

[R36] Li K-C. 1992. “On Principal Hessian Directions for Data Visualization and Dimension Reduction: Another Application of Stein’s Lemma.” Journal of the American Statistical Association 87, no. 420: 1025–1039.

[R37] Liang F. 2005. “Bayesian Neural Networks for Non-Linear Time Series Forecasting.” Statistics and Computing 15: 13–29.

[R38] Liang F, Jia B, Xue J, Li Q, and Luo Y. 2018. “An Imputation-Regularized Optimization Algorithm for High-Dimensional Missing Data Problems and Beyond.” Journal of the Royal Statistical Society. Series B, Statistical Methodology 80, no. 5: 899–926. 10.1111/rssb.12279 PMC6533005 31130816

[R39] Liang F, Li Q, and Zhou L. 2018. “Bayesian Neural Networks for Selection of Drug Sensitive Genes.” Journal of the American Statistical Association 113: 955–972. 10.1080/01621459.2017.1409122 PMC6660200 31354179

[R40] Liang S, Huang W-H, and Liang F. 2021. “Sufficient Dimension Reduction With Deep Neural Networks for Phenotype Prediction.” In Proceedings of the 3rd International Conference on Statistics: Theory and Applications, 134. Avestia Publishing.

[R41] Liang S, Sun Y, and Liang F. 2022. “Nonlinear Sufficient Dimension Reduction With a Stochastic Neural Network.” NeurIPS.

[R42] Loh P, and Wainwright M. 2017. “Support Recovery Without Incoherence: A Case for Nonconvex Regularization.” Annals of Statistics 45, no. 6: 2455–2482.

[R43] Louizos C, Shalit U, Mooij JM, Sontag DA, Zemel RS, and Welling M. 2017. “Causal Effect Inference With Deep Latent-Variable Models.” Neural Information Processing Systems 30: 6446–6456.

[R44] MacKay DJC. 1996. “Bayesian Non-Linear Modeling for the Prediction Competition.” In Maximum Entropy and Bayesian Methods: Santa Barbara, California, U.S.A., 1993, edited by Heidbreder GR, 221–234. Springer Netherlands.

[R45] Marinazzo D, Pellicoro M, and Stramaglia S. 2008. “Kernel-Granger Causality and the Analysis of Dynamical Networks.” Physical Review E 77: 056215. 10.1103/PhysRevE.77.056215 18643150

[R46] Neal RM. 1996. Bayesian Learning for Neural Networks. Springer.

[R47] Nielsen S. 2000. “The Stochastic Em Algorithm: Estimation and Asymptotic Results.” Bernoulli 6: 457–489.

[R48] Nilsson J, Sha F, and Jordan MI. 2007. “Regression on Manifolds Using Kernel Dimension Reduction.” In Proceedings of the 24th International Conference on Machine Learning, 697–704. ACM.

[R49] Noh H, You T, Mun J, and Han B. 2017. “Regularizing Deep Neural Networks by Noise: Its Interpretation and Optimization.” ArXiv, abs/1710.05179.

[R50] Pearl J. 2009. Causality: Models, Reasoning, and Inference. 2nd ed. Cambridge University Press.

[R51] Poggio T, Mhaskar H, Rosasco L, Miranda B, and Liao Q. 2017. “Why and When Can Deep–but Not Shallow-Networks Avoid the Curse of Dimensionality: A Review.” International Journal of Automation and Computing 14, no. 5: 503–519.

[R52] Radhakrishnan A, Beaglehole D, Pandit P, and Belkin M. 2024. “Mechanism for Feature Learning in Neural Networks and Backpropagation-Free Machine Learning Models.” Science 383: 1461–1467. 10.1126/science.adi5639 38452048

[R53] Robins JM, Rotnitzky A, and Zhao LP. 1994. “Estimation of Regression Coefficients When Some Regressors Are Not Always Observed.” Journal of the American Statistical Association 89: 846–866.

[R54] Rosenbaum PR. 2002. Observational Studies. (2nd edition) ed. Springer.

[R55] Rubin DB. 1974. “Estimating Causal Effects of Treatments in Randomized and Nonrandomized Studies.” Journal of Educational Psychology 66: 688–701.

[R56] Salakhutdinov R, and Hinton G. 2009. “Deep Boltzmann Machines.” In Proceedings of the International Conference on Artificial Intelligence and Statistics, 448–455. JMLR.org.

[R57] Siggiridou E, and Kugiumtzis D. 2021. “Dimension Reduction of Polynomial Regression Models for the Estimation of Granger Causality in High-Dimensional Time Series.” IEEE Transactions on Signal Processing 69: 5638–5650.

[R58] Sims CA. 1980. “Macroeconomics and Reality.” Econometrica 48, no. 1: 1–48.

[R59] Smolensky P. 1986. “Information Processing in Dynamical Systems: Foundations of Harmony Theory.” In Parallel Distributed Processing: Explorations in the Microstructure of Cognition, edited by Rumelhart DE and McClelland JL, vol. 1, 194–281. MIT Press.

[R60] Sun Y, and Liang F. 2022. “A Kernel-Expanded Stochastic Neural Network.” Journal of the Royal Statistical Society, Series B 84, no. 2: 547–578.

[R61] Sun Y, and Liang F. 2027. “Uncertainty Quantification for Large-Scale Deep Neural Networks via Post-StoNet Modeling.” Statistica Sinica 37, no. 4.

[R62] Sun Y, Song Q, and Liang F. 2022a. “Consistent Sparse Deep Learning: Theory and Computation.” Journal of the American Statistical Association 117, no. 540: 1981–1995. 10.1080/01621459.2021.1895175 PMC10027379 36945326

[R63] Sun Y, Song Q, and Liang F. 2022b. “Learning Sparse Deep Neural Networks With a Spike-And-Slab Prior.” Statistics & Probability Letters 180: 109246. 10.1016/j.spl.2021.109246 PMC8570537 34744226

[R64] Suzuki T, and Sugiyama M. 2010. “Sufficient Dimension Reduction via Squared-Loss Mutual Information Estimation.” In Proceedings of the Thirteenth International Conference on Artificial Intelligence and Statistics, 804–811. JMLR Workshop and Conference Proceedings. 10.1162/NECO_a_00407 23272920

[R65] Tang Y, and Li B. 2026. “Belted and Ensembled Neural Network for Linear and Nonlinear Sufficient Dimension Reduction.” Journal of the American Statistical Association 121, no. 554: 1244–1255.

[R66] Tank A, Covert I, Foti N, Shojaie A, and Fox EB. 2022. “Neural Granger Causality.” IEEE Transactions on Pattern Analysis and Machine Intelligence 44, no. 8: 4267–4279. 10.1109/TPAMI.2021.3065601 PMC9739174 33705309

[R67] Tibshirani R. 1996. “Regression Shrinkage and Selection via the Lasso.” Journal of the Royal Statistical Society. Series B, Statistical Methodology 58: 267–288.

[R68] Wu H-M. 2008. “Kernel Sliced Inverse Regression With Applications to Classification.” Journal of Computational and Graphical Statistics 17, no. 3: 590–610.

[R69] Xia Y. 2007. “A Constructive Approach to the Estimation of Dimension Reduction Directions.” Annals of Statistics 35, no. 6: 2654–2690.

[R70] Xia Y, Tong H, Li W, and Zhu L-X. 2002. “An Adaptive Estimation of Dimension Reduction Space.” Journal of the Royal Statistical Society. Series B, Statistical Methodology 64, no. 3: 363–410.

[R71] You Z, Ye J, Li K, and Wang P. 2018. “Adversarial Noise Layer: Regularize Neural Network by Adding Noise.” In 2019 IEEE International Conference on Image Processing (ICIP), 909–913. IEEE.

[R72] Yu T, Yang Y, Li D, Hospedales TM, and Xiang T. 2021. “Simple and Effective Stochastic Neural Networks.” AAAI.

[R73] Zhang C-H. 2010. “Nearly Unbiased Variable Selection Under Minimax Concave Penalty.” Annals of Statistics 38: 894–942.

[R74] Zhang M, Sun Y, and Liang F. 2023. “Sparse Deep Learning for Time Series Data: Theory and Applications.” NeurIPS.

[R75] Zhao P, and Yu B. 2006. “On Model Selection Consistency of Lasso.” Journal of Machine Learning Research 7: 2541–2563.

[R76] Zhou W, Bai S, Yu S, Zhao Q, and Chen B. 2024. “Jacobian Regularizer-Based Neural Granger Causality.” In Proceedings of the 41st International Conference on Machine Learning. ICML.

